# The New Environmental Health in Australia: Failure to Launch?

**DOI:** 10.3390/ijerph18041402

**Published:** 2021-02-03

**Authors:** James C. Smith, Harriet Whiley, Kirstin E. Ross

**Affiliations:** Environmental Health, College of Science and Engineering, Flinders University, Bedford Park 5042, Australia; jim@jamescsmith.com.au (J.C.S.); harriet.whiley@flinders.edu.au (H.W.)

**Keywords:** environmental health, practice, policy, New Environmental Health

## Abstract

Background: The New Environmental Health is an approach to environmental health adopted in 1999. The new approach was in response to emerging health risks from the pressures that development placed on the environment, climate change, and increasing vulnerabilities of local communities. The new approach heralded a change in perception and roles within environmental health. Twenty years on, it seems these changes have not been embraced by local government. Methods: To determine whether this was the case, we assessed the use of the term “environmental health” in local government annual reports, and where environmental health functions sit within the organisational structure of councils. Results: We found that the New Environmental Health has not been adopted by councils and environmental health relates solely to the delivery of statutory services and legislative compliance. Conclusions: One result of this is local environmental health practitioners, who constitute the major health protection capability of councils, are defined by the narrow legislative obligations imposed on councils. This represents a significant lost opportunity as public health is not protected in the way that was envisaged with the adoption of the New Environmental Health.

## 1. Introduction

In 1999, the Australian National Environmental Health Strategy introduced the “New Environmental Health”, which was about creating and maintaining environments that promote good public health rather than the health or protection of the environment, or improving living conditions and controlling epidemic diseases of the 19th and 20th century [1]. At the international level, the World Health Organization (WHO) had defined environmental health as:

“… those aspects of human health, including quality of life, that are determined by chemical, physical, biological, social and psychosocial factors in the environment. It also refers to … assessing, correcting, and preventing those factors in the environment that can potentially affect adversely the health of present and future generations” (World Health Organization 1993, cited in Cromar et al. [2].

The Strategy stated that the traditional role of practitioners, with its strong focus on enforcement and monitoring of legislative requirements, would need to change to meet future environmental health management demands [1]. The National Environmental Health Council (enHealth) was established as the peak environmental health advisory group in Australia with membership including representatives from the Australian Local Government Association (ALGA) and the Australian Institute of Environmental Health (AIEH), which was the representative body for environmental health officers mainly employed in local government. EnHealth developed the National Environmental Health Strategy–Implementation Plan which contained roles and actions for both ALGA and AIEH in workforce and professional development [3].

Twenty years on and “environmental health” is described by the WHO as still being concerned with addressing those physical, chemical, and biological factors adversely affecting health, and with practices requiring the assessment and control of those factors with the aim of preventing disease, and creating health supporting environments [4]. The Australian government has adopted a definition stating that “environmental health involves those aspects of public health concerned with factors, circumstances, and conditions in the environment or surroundings of humans that can exert an influence on health and well-being” and widening practice to address “… emerging health risks arising from the pressures that human development places on the environment” [5]. 

In its 2016–2020 Strategic Plan, enHealth introduced the concept of “environmental public health” and defined it as sitting within “… the broader scope of health protection functions, which seek to reduce the likelihood and minimise the consequence of both known and unknown risks that arise, … from … infectious diseases, chemicals, radiological agents, natural disasters and other mass casualty events …” [5]. The New Environmental Health still retained its focus on creating and maintaining environments which promote good public health or, as expressed by WHO, creating health supporting environments. 

Local councils in Australia have a longstanding role in environmental health with much of the role being enshrined in public health legislation [6,7]. However, as has been mentioned, The New Environmental Health is about creating and maintaining environments conducive to public health and going beyond minimum levels of health protection demanded by legislation and responding to environmental hazards from demanding lifestyles, increasing urbanisation, globalisation, and climate change [1,8]. This is quite different to what is considered the “old” view of environmental health with its restricted focus on epidemic diseases of the 19th century [1,9].

Local councils exist to achieve goals demanded by their community, or by legislation delegated by state parliaments. The achievement of these goals requires organisation of resources and management processes into an effective organisational structure [10]. Organisational structure is defined as the distribution of units and positions within the organisation, or the grouping of these, and their systematic relationships to each other, with each subsystem structure having a set of functions associated with it [10,11]. Governments in Australia have operated with hierarchical structures since federation [12], and this model emphasises, amongst other things, departmentalisation [13], which is a fundamental characteristic of an organisational structure [14]. Functional departmentalisation involves grouping similar activities [10], or similar skills and expertise [14], or the performance of similar functions, into the same department [13,15]. 

A key corporate management process for councils is their annual plan describing the council’s strategic objectives and strategies, and subsequent reporting describing its performance for the year [16].

As twenty years have elapsed since the introduction of the New Environmental Health heralding a new environmental health approach and practice, we wanted to assess if these changes had been embraced by local government. To do this, we assessed how the term “environmental health” has been used in local government annual reports, and how environmental health functions have been departmentalised within the organisational structure of councils. Given that many countries subscribe to the WHO definition of environmental health, the implementation of the New Environmental Health concept in Australia has international implications and significance [17].

## 2. Materials and Methods 

Thirty-six councils were randomly selected from across Australia (there are 537 councils in Australia [18]). These included 20 metropolitan councils (with a rural, remote, and metropolitan area (RRMA) classification of 1 or 2) and 16 rural and remote councils (with an RRMA of 3 to 7) [19]. The annual reports and organisational information were obtained from their respective websites between September 2018 and October 2019. A two-part content analysis was conducted of each report. The first part consisted of a search for “environmental health”, the number of times it occurred and its context, with the results recorded and tabulated for each council. The second part consisted of examining councils’ organisational charts, locating the environmental health functions, and tabulating these. A categorisation and collation process was conducted based on the terms used by councils to identify and describe the department. A similar process was conducted for the next management level (subsystem) down in each department to identify other functions associated with environmental health activities.

Seven peak local government body websites, two online local government employment sites (Australian Local Government Job Directory (https://www.job-directory.com.au) and LG Assist—Australian Local Government Employment Website (https://www.lgassist.com.au)), and three advertised senior Environmental Health Officer positions were also examined to ascertain the perceptions of environmental health. The seven local government websites examined were the Australian Local Government Association (ALGA) and the state representative bodies including the Western Australian Local Government Association (WALGA), Local Government Association of South Australia (LGA of SA), Municipal Association of Victoria (MAV), Local Government Association of Tasmania (LGAT), Local Government New South Wales (LGNSW), and Local Government Association Queensland (LGAQ).

## 3. Results

Of the 36 councils examined, there were 9 from NSW; 3 from Queensland; 9 from South Australia; 3 from Tasmania; 10 from Victoria; and 2 from Western Australia. An examination of the 2017–2018 annual reports showed that 11 reports did not mention the term “environmental health”. Of the remaining reports, the term was used on 85 occasions; 53 in relation to organisational contexts; 29 in relation to a service context; and 4 in relation to a legislative context. No annual report provided a description or definition of environmental health. There were no differences observed in the recognition of environmental health between metropolitan or rural and remote councils.

Table 1 outlines the results of the categorisation process and shows environmental health was located in departments with a focus on service (12), planning (9), development (7) strategy (4), and environment (2). There were two reports where departmentalisation was unclear. Within the service categorisation, there were three occasions where environmental health was part of corporate services, three where it was part of development services, and the remaining six spread in other areas. Within the planning categorisation, environmental health was located within mainly development, environment, and city planning. Within the development categorisation, environmental health was located within community, city, and infrastructure. 

The names given to the sub-departmental functional areas were examined excluding six councils where activities were managed directly by an executive. Of the remaining 30 councils, 19 had management unit titles that included the terms “regulation”, “compliance”, “inspectorial” or “statutory”. 

Table 2 presents these management unit titles, and any details describing the activities undertaken. In all but one council, the range of activities documented by councils were described using regulatory terms with no mention of activities associated with the New Environmental Health, that is, activities relating to lifestyle, increasing urbanisation, globalisation or climate change issues. The eleven management unit titles not so described are presented in Table 3 with information describing the activities undertaken. Terms relating to regulatory activities were used in all but one of the activity descriptions. Thus, 29 councils located environmental health with the regulatory compliance functions of the council.

The search of local government association websites revealed no references to “environmental health” other than references to job vacancies on one site, and a legislation reference on another. 

Table 4 outlines the position objectives and first mentioned key responsibility for the advertised senior environmental health positions.

The first position description’s objective and responsibilities focussed on providing services to protect community health in accordance with statutory requirements. A similar theme can be seen in the second position description, that is, high standards of operations through inspection, education, monitoring, and enforcement services. The third position description focussed on ensuring regulatory compliance standards as both the means and outcome of the position.

## 4. Discussion

The term “environmental health” was not mentioned in eleven annual reports and, where it is mentioned, it was in reference to services or environmental health staff. This snapshot demonstrates that the New Environmental Health seems not have been adopted/implemented. This, with the absence of a definition or description of “environmental health” in any report and on local government peak body websites, suggests that councils are unfamiliar with the term espoused in the 1999 National Environmental Health Strategy, or by the World Health Organization [4] and EnHealth [5]. This lack of familiarity is of particular interest as ALGA and the AIEH were members of the original enHealth (the National Environmental Health Council) with responsibilities under its implementation plan; however, neither organisation is a member of the current Environmental Health Standing Committee (enHealth). The Committee’s website states that it “… works with Australian local government associations, … and non-government organisations such as Environmental Health Australia, Public Health Association of Australia and Choice” [20]. Further, it is noted that the National Environmental Health Strategy 2016–2020 is the Committee’s Strategic Plan, and responsibility for its implementation rests solely with the Committee. From the inception of The New Environmental Health thinking in 1999 to now, there has been a change in local government’s relationship with the Committee and its connection with environmental health policy development and is reliant on effective consultation by the Committee. This may explain, in part, the lack of familiarity with The New Environmental Health by local government. 

The New Environmental Health was concerned with achieving health supporting environments and therefore needed to be thought of as a cause, or a field, and not as a profession or a discipline, with a scope that is much wider than the traditional perspectives of environmental health [21] and requires the engagement of a wide array of personnel [6,22]. This need for a change in conception was identified in the United Kingdom, where there were tensions with local authorities meeting narrow regulatory requirements rather than leading on a wider public health policy and practice [23]. A similar observation was made by Reynolds and Wills [24] who advanced the concept of “environmental healthness” where solutions are generated for problems from a “holistic public health position”. 

It is seen in Table 1 that the organisational location of environmental health can be in any department, although those departments focussing on services, planning and development account for the majority of locations. This diversity of locations suggests that local government does not have a common understanding of how environmental health makes a contribution to corporate objectives. However, the information contained in Table 2 and Table 3 shows that this contribution, in 29 of 36 councils, is in the delivery of regulatory services with the associated activities of inspections, surveillance, and enforcement and is usually alongside other regulatory services. As noted, there was no mention of activities associated with the New Environmental Health. It is not suggested that such activities are not undertaken by councils, but it appears these activities are not associated with councils’ concepts of environmental health. The delivery of statutory services as the priority of environmental health is corroborated further by the three position descriptions for senior environmental health staff in three different councils. 

There were 85 references to “environmental health” in annual reports, and 53 of these were about regulatory services or environmental health officers, indicating that councils associate environmental health with EHOs and their activities. It appears that local government perceives environmental health in terms of the narrow and traditional compliance and enforcement role consistent with the observations made by Dhesi and Stewart [23]. Interestingly, Wright [21] made the observation that local environmental health officers define their professional role by the legislative obligations imposed on councils:

“… it appears to me that environmental health practitioners, unlike other professionals in say, law, medicine or engineering define themselves by reference to the organisation in which they find themselves rather than by reference to their professional competencies.” 

A critical consequence is that environmental health practitioners, and the critical role they play in public health protection, are hidden behind the “organisational curtain” of local government and are thus invisible to the general public. This may detrimentally affect future funding and support as political agendas and public policy can be influenced by public opinion [25]. In addition, the lack of recognition hinders workforce recruitment and efforts to secure a sustainable workforce to future proof public health protection [26].

In contrast to The New Environmental Health, The New Public Health, with its foundation in the Ottawa Charter for Health Promotion [27,28], seems to have had more impact. “The [Ottawa} Charter recognises the importance of social, economic and physical environmental factors in shaping people’s experiences of health” [29]. Subsequent, international conferences on health promotion (for example, Sundsvall in 1991) linked the health of the physical environment explicitly with health promotion [29,30]. 

The ideas of the New Public Health found expression in the 1986 Healthy Cities Program [29,31], which has been adopted in many countries including Australia [32], and were taken up specifically by the Victorian and South Australian governments in the 1980s [29] and, subsequently, extended into the local government sector. In Victoria, The New Public Health was the basis for municipal public health plans and the healthy localities project as local government began to view itself “… as a locus where the rhetoric of The New Public Health … could be realised” [33]. Similarly, in the 1990s, councils in Queensland adopted municipal public health planning based on the WHO healthy cities movement [34]. In 2001, the Victorian Department of Human Services developed the Environments for Health framework to assist the development of public health plans by councils. This framework recognises that health and wellbeing are affected by social, built, natural, and economic dimensions. The strong link between built environment and health and wellbeing is identified, and linkages made with the Key Parameters for Healthy Cities [35]. This framework was taken up by Western Australia and is directly applicable for the development of local public health plans in South Australia [36]. It is noted that the planning guide developed by the LGA of SA referred to environmental health as a common council business activity in the social environmental dimension [36].

Although the Framework refers to the National Environmental Health Strategy, it does so in the context of a national strategy with action only at the national level and with no reference to The New Environmental Health approach. 

It appears that although environmental health is concerned with creating health supporting environments, it was not adopted by local councils into its planning processes, whereas the Environments for Health framework based in The New Public Health was adopted actively in many councils and was facilitated by its peak bodies. Inexplicably, at the local government level, there is no policy connection between The New Environmental Health and The New Public Health, even though both have a focus on healthy environments. Any future review of the Framework should link environmental health policy into the local public and environmental health planning framework as was first envisaged in the 1999 National Environmental Health Strategy [1]. 

## 5. Conclusions

This study demonstrates that the contemporary understanding of environmental health in local government does not reflect the New Environmental Health concept that was launched in Australia 20 years ago. The current understanding of environmental health remains one that is narrow and focussed on legislative compliance and does not reflect the ambitious diversity inherent in the New Environmental Health concept. The national vision for environmental health has not been disseminated into local government. 

The reasons for councils not adopting The New Environmental Health thinking seems to lie with the removal of local government and the AIEH (now EHA) from membership of enHealth and Environmental Health Standing Committee, the lack of advocacy by peak bodies and respective state government health departments for the new thinking and its implications for councils, and the absence of linkages between policy initiatives associated with the New Public Health movement and approach of The New Environmental Health. One result of the failure to adopt the new approach is for local environmental health practitioners, who constitute the major health protection capability of councils, continue being defined by the narrow legislative obligations imposed on councils. Future implementation strategies should focus on ensuring strong participatory, communication, and support links between stakeholders to ensure the vision that is conceived at the national level is implemented at the local level.

The New Environmental Health thinking is concerned with ensuring the protection of local communities’ health from known and unknown factors in the built and natural environments. This includes the challenges of climate change and the impact this will have on recognised prerequisites for health such as shelter, safe and secure food and water, and a stable ecosystem; and the challenges with changes in microorganisms as seen with the COVID-19 pandemic and the resulting health, social, and economic impacts on local communities. These challenges demand the appropriate systematic investigation of local environmental and public health risk factors, risk assessment of these factors, and the development of risk mitigation strategies that are integrated with the local public health planning frameworks adopted by councils. The skills and knowledge required to undertake these risk management activities reside in the local university qualified environmental health workforce and, by utilising and integrating these with the public health planning framework, councils have an important resource to assist the development of a supportive environment that will protect and promote good public health into the future. 

## Figures and Tables

**Table 1:** Location of environmental health services by department.

Department	Department Categorisation	Categorisation Summary
Director of Community Development	Development	Community × 3 City × 2 Infrastructure × 2
GM City Development	Development	
Infrastructure, Works, and Development	Development	
City Development	Development	
Development and Community	Development	
Director Community Development	Development	
Infrastructure and Development	Development	
City Development	Development	
	7	
Planning and Environment	Planning	Environment × 2 Development × 3 Place × 1 Economic × 1 City × 2
Planning and Environment	Planning	
Director Planning and Place	Planning	
City Planning, Design, and Amenity	Planning	
Planning and Development	Planning	
City Planning	Planning	
Community, Environment, and Planning	Planning	
Planning and Economic Development	Planning	
Planning and Development	Planning	
	9	
Corporate Services	Services	Corporate × 3 Development × 3 Operations × 1 Planning & Regulatory × 1 Customer Engagement × 1 Planning & Corporate Services × 1 City × 1 Land & Environment × 1
City Services	Services	
Planning and Regulatory Services	Services	
Development Services	Services	
Development Services	Services	
Development Services	Services	
Land and Environment Services	Services	
Director Corporate, Business, and Financial Services	Services	
General Manager Operations	Operations	
Customer Engagement and Planning Services	Services	
Director Corporate Services	Services	
Director Planning and Corporate Services	Services	
	12	
Environmental and Inspectorial	Environment	
City Environment	Environment	
	2	
City Strategy Division	Strategy	
Director of City Sustainability and Strategy	Strategy	
Director Strategy and Sustainability	Strategy	
City Life	Strategy	
	4	
Not designated		
Not designated		
	2	
Total	36	

**Table 2:** Environmental health and sub-departmental functions.

Management Unit	Nominated Activities/Functions
Certification and Compliance	No details provided
Certification and Compliance	No details provided
Health, Building and Regulatory Services	Regulatory environmental health and building services
Compliance	Health, building, environmental compliance, rangers, and parking
Waste and Compliance Services	No details provided
Regulatory Services	Compliance, education and enforcement functions related to public health, animal management, fire prevention, local laws, parking, litter, and planning
Regulatory Services	Reduction of unlawful activity related to building compliance, food health safety, and parking
Health, Environment, and Regulatory Services	Regulatory services, health and immunisation, environment, and waste
Regulatory Services	Environmental health services, planning and building, economic and tourism development
Health and Compliance	Environmental health, regulations, compliance, and immunisation
Development and Regulatory Services	Inspection, regulation, and control
Regulatory Services	Town Planning and Development, Building Permit Authority, Plumbing Assessment and Inspections, Environmental Health Services, Animal Control
Environmental and Building Compliance	All relevant health, safety and environmental matters meet appropriate standards—food safety, public health, environmental and development non-compliance
Environmental Compliance	Public health, safety and the natural environment, rangers
Compliance and Parking	Statutory enforcement services, including animal management, local law enforcement, food safety and school crossing management
Regulation and Enforcement	Reactive inspection programs based on customer requests in addition to targeted enforcement programs aimed at improving awareness, and where necessary, undertaking enforcement action
Regulatory Services	Community Safety, Statutory Planning, and Regulatory
Environmental and Inspectorial	Animal management, waste management, development, water management, general inspections, environmental health
Compliance	Environmental Health Officer and General Inspector
Planning and Statutory Services	Planning, Building and Environmental Health, Finance and Risk, Information Technology, Rates, Enforcement

**Table 3:** Environmental health and associated sub-system functions.

Management Unit	Nominated Activities/Functions
Community and Environmental Health	Undertake food inspections and education to make sure food for sale in the City is safe
Environmental Health	Municipal Inspection, Land Use planning, building compliance, Environmental Health, Cemetery Management, Parking and Weed Management
Environmental Health	Community safety in public facilities, including public pools, public buildings. Environmental health activities included responding to noise and other nuisance complaints, routine surveillance and audits of food premises and temporary events
Environment	The Environmental Health Unit is responsible for implementing policies and monitoring compliance through inspections and investigations
Community Amenity	Public health unit and community laws unit (parking control; animal management; administering and enforcing Council’s local laws)
Health, Environment and Waste	Environmental health, including food business licensing, public health inspections and immunisation; waste and recycling
Environment and Planning	Water and Wastewater; Environmental Health; Planning; Local Laws; Organisational Sustainability; Civil Operations; Public Space; and Maintenance and Construction
Development	Planning, building, environmental health and General Inspection
Planning, Building, and Health	Statutory Planning and Regulations; Strategic Land Use Planning; Economic Development; Events; Tourism; Airfields; Environmental Management; Building Surveying; Environmental Health; Community Services; Emergency Management and Fire Prevention; Local Laws
City Development	Planning, Building, Health Protection, and Statutory Planning
Healthy Environments	Environmental Health, Waste, Parks and Gardens, Climate Change, Natural Environment

**Table 4:** Position objectives of advertised senior environmental health officer.

Position Title	Position Objective	Key Position Responsibilities and Duties
Environmental Health Coordinator	To lead, coordinate, and continuously improve the provision of quality environmental health services that protects and enhances community health and wellbeing	Coordinate, motivate, and engage the Environmental Health and Building Teams to deliver outputs and outcomes in accordance with relevant statutory requirements.
Environmental Health Coordinator	Provide leadership, direction, and oversight to ensure the ongoing high standards of the activities and operations of the Environmental Health team. Supervise the inspection, monitoring, regulation, education, and enforcement services that protect and improve the health and wellbeing of the community and environment	Lead, motivate, develop, and support the Environmental Health team to achieve operational and strategic goals and maintain high standards of practice.
Team Leader Compliance and Building Regulation	To supervise and actively participate in the activities of the team ensuring regulatory compliance standards are met in relation to fire safety, on-site sewage management, swimming pool barriers, pollution events, petroleum storage facilities, public health, food safety, and the like	Provide day-to-day guidance and technical/professional expertise to team members of the compliance team.

## Data Availability

Not applicable.
